# Effectiveness of community-based burden estimation to achieve elimination of lymphatic filariasis: A comparative cross-sectional investigation in Côte d’Ivoire

**DOI:** 10.1371/journal.pgph.0000760

**Published:** 2022-08-31

**Authors:** Hope Simpson, Daniele O. Konan, Kouma Brahima, Jeanne d’Arc Koffi, Saidi Kashindi, Melissa Edmiston, Stefanie Weiland, Katherine Halliday, Rachel L. Pullan, Aboulaye Meite, Benjamin Guibehi Koudou, Joseph Timothy

**Affiliations:** 1 Department for Disease Control, London School of Hygiene and Tropical Medicine, London, United Kingdom; 2 Centre for Global Health Research, Brighton and Sussex Medical School, Brighton, United Kingdom; 3 Centre Suisse de Recherches Scientifiques en Côte d’Ivoire, Abidjan, Côte d’Ivoire; 4 Ministère de la Sante et de l’Hygiène Publique, Programme national de lutte contre les Maladies Tropicales Négligées à Chimiothérapie Préventive (PNLMTN-CP) en Côte d’Ivoire, Abidjan, Côte d’Ivoire; 5 American Leprosy Missions, Greenville, South Carolina, United States of America; 6 AIM Initiative, Accra, Ghana; Fundacao Oswaldo Cruz, BRAZIL

## Abstract

For lymphatic filariasis (LF) elimination, endemic countries must document the burden of LF morbidity (LFM). Community-based screening (CBS) is used to collect morbidity data, but evidence demonstrating its reliability is limited. Recent pilots of CBS for LFM alongside mass drug administration (MDA) in Côte d’Ivoire suggested low LFM prevalence (2.1–2.2 per 10,000). We estimated LFM prevalence in Bongouanou District, Côte d’Ivoire, using a comparative cross-sectional design. We compared CBS implemented independently of MDA, adapted from existing Ministry of Health protocols, to a population-based prevalence survey led by formally trained nurses. We evaluated the reliability of case identification, coverage, equity, and cost of CBS. CBS identified 87.4 cases of LFM per 10,000; the survey identified 47.5 (39.4–56.3; prevalence ratio [PR] 1.84; 95% CI 1.64–2.07). CBS identified 39.7 cases of suspect lymphoedema per 10,000; the survey confirmed 35.1 (29.2–41.5) filarial lymphoedema cases per 10,000 (PR 1.13 [0.98–1.31]). CBS identified 96.5 scrotal swellings per 10,000; the survey found 91.3 (83.2–99.8; PR 1.06 [0.93–1.21]); including 33.9 (27.7–38.8) filarial hydrocoele per 10,000 (PR of suspect to confirmed hydrocele 2.93 [2.46–3.55]). Positive predictive values for case identification through CBS were 65.0% (55.8–73.5%) for filarial lymphoedema; 93.7% (89.3–96.7%) for scrotal swellings; and 34.0% (27.3–41.2%) for filarial hydrocoele. Households of lower socioeconomic status and certain minority languages were at risk of exclusion. Direct financial costs were $0.17 per individual targeted and $69.62 per case confirmed. Our community-based approach to LFM burden estimation appears scalable and provided reliable prevalence estimates for LFM, scrotal swellings and LF-lymphoedema. The results represent a step-change improvement on CBS integrated with MDA, whilst remaining at programmatically feasible costs. Filarial hydrocoele cases were overestimated, attributable to the use of case definitions suitable for mass-screening by informal staff. Our findings are broadly applicable to countries aiming for LF elimination using CBS. The abstract is available in French in the S1 File.

## Introduction

Lymphatic filariasis (LF) is a mosquito-borne disease caused by the filarial nematodes *Wuchereria bancrofti*, *Brugia malayi* and *Brugia timori* [1], estimated to infect 51 million people globally [2]. Progression of the infection to chronic disease is associated with progressive damage to the lymphatic system, which can lead to irreversible swelling and acute attacks of dermato-lymphangio-adenitis (ADLA). The two most overt manifestations of chronic LF are lymphoedema- caused by accumulation of lymph fluid in the soft tissue, generally affecting a limb or breast- and hydrocoele, caused by accumulation of lymph fluid inside the scrotal sac [3]. Although the number of people affected by lymphatic filariasis morbidity (LFM) is difficult to quantify (data being hard to collect and predictions unreliable), the burden of disease is undoubtedly substantial—estimates from 2012 suggested 19.4 million men with filarial hydrocoele and 16.7 million people with lymphoedema globally [4]. Disability‐adjusted life year (DALY) estimates place LF as the second leading cause of disability due to parasitic diseases [5].

Since 1997, LF has been targeted for elimination as a public health problem, with efforts coordinated by WHO under the Global Programme to Eliminate Lymphatic Filariasis (GPELF) [6]. The elimination strategy consists of two components: interruption of transmission through mass drug administration (MDA) in endemic areas, and alleviation of suffering through morbidity management and disability prevention (MMDP) [6]. Verification of elimination depends on the sustained reduction of prevalence to below 1%, documentation of lymphoedema and hydrocoele case numbers, and the readiness and quality of MMDP services in health facilities in endemic areas [7]. While MDA has helped reduce LF infection prevalence, many people remain affected by lymphoedema and hydrocoele and require these MMDP services. However, most endemic countries lack estimates of the numbers of people affected and their distribution. There is no standardised mechanism for the collection of data on LFM, but due to resource limitations and barriers to care-seeking at health facilities [8–10], case detection in many endemic settings depends upon existing community health infrastructure [11]. Community-based interventions, particularly MDA, have been central to NTD control, enabling access to marginalised communities and higher intervention coverage [12–14], and have been successfully used for case-finding in Guinea worm eradication [15]. The suitability of these staff to identify and refer cases of more subtle, chronic NTD morbidity is less certain, however. Community-based screening (CBS) of LFM during MDA is used in Burkina Faso, Ghana and Malawi [16] and other countries, but is understood to substantially underestimate true case numbers [17].

In Côte d’Ivoire, where LF is endemic in 99 of 103 health districts and infection prevalence is predicted to exceed 1% [2], the NTD Control Programme (NTDP) plans to estimate LFM burden using CBS to guide service delivery and achieve LF elimination. The system will be scaled up gradually, providing opportunity to iteratively tailor and strengthen it. CBS through MDA piloted in 2020 identified 20 cases of lymphoedema and 9 of hydrocoele in the district of Lakota (population > = 15 years old 132,216), and 34 cases of lymphoedema and 17 of hydrocoele in the district of Divo (population > = 15 years old 248,613) [18, 19]. We aimed to improve this existing approach by de-coupling case finding from MDA, improving and strengthening the CDD training programme, and implementing simple methodological adaptations. We evaluated the effectiveness of this strengthened, standalone strategy against a population-based prevalence survey led by nurses specially trained in LFM diagnosis. We also conducted a process evaluation during implementation to understand operational factors affecting performance including diagnostic reliability, household coverage, equity, and financial cost.

## Materials and methods

### Setting

The study was conducted in the health district of Bongouanou, in the Moronou Region of centre-east Côte d’Ivoire (S1 Fig). The district is LF-endemic, with evidence from geospatial and models and a community-based survey using ICT cards in 2012 suggesting high prevalence of parasitaemia [2, 20, 21], but had not previously been surveyed for LFM. The landcover is primarily Guinean forest-savanna mosaic and the main industry is cacao and coffee farming [22]. Compared to other districts in the region, the infant mortality rate is low, public and private-sector salaries are high, and there are high levels of access to piped water [22]. The district population used for health operation purposes including MDA, is 169,999, the population > = 15 years old is 94,319 and the male fraction is 0.50 [18]. The district is serviced by 24 health facilities: one general hospital, four urban health centres, eight rural health centres, and ten dispensaries. The area covered by each health facility is referred to as a health area, and populations within these health areas range from 800–26,513.

### Study design and participants

We did a comparative cross-sectional study of methods to estimate LFM prevalence, and follow the STROBE guidelines for reporting of cross-sectional studies (see S1 Checklist). In the first phase of data collection, between 22^nd^ and 26^th^ February 2021, all LF-MDA community drug distributors (CDDs) completed a stand-alone, exhaustive door-to-door case search for leg swellings (suspect lymphoedema) and scrotal swellings (suspect hydrocoele) covering the entire district population aged 15 years and over. In the second phase of data collection, conducted immediately after (15^th^ March- 17^th^ June 2021), nurses specially trained in diagnosis of LFM conducted a population-based prevalence survey. Results from the CDD-led screening were compared to those from the population-based prevalence survey.

For the population-based prevalence survey, we used a stratified two-stage cluster-based design with strata based upon health areas, primary sampling units (PSUs) defined as CDD zones and secondary sampling units (SSUs) as households [23]. Total PSUs selected within strata was determined using proportional allocation. PSUs were selected using simple random sampling without replacement, due to absence of population data at CDD zone level. All individuals aged 15 years and older in selected PSUs were eligible for participation. Using a standard sample size calculation [23] assuming a prevalence of 5 cases per 1,000 population, a participation rate of 95%, design effect of 5.95, and applying a finite population correction factor for the population of Bongouanou district, we calculated that 12,217 participants needed to be examined to estimate LFM prevalence with an absolute precision of 0.003.

To assess diagnostic reliability of CBS, CDDs recruited all cases they had identified and who had not been examined through the population-based prevalence survey, for re-examination by nurses at a central location after completion of surveys within PSUs.

### Procedures

#### Co-development of toolkit for community-based screening

The CDD toolkit, including the training of trainers guide, slide-deck, job-aid and photobook, was developed by the project team before being presented to a representative of the NTDP (BK) for further revisions. The draft materials were then extensively reviewed and modified at a 3-day revisions workshop held in Yamoussoukro, attended by the NTDP, members of the project team, and the team who had been involved in pilot CBS implemented by the MoH NTD programme in 2020 (members of the district health team, one CDD, and partners). The revised toolkit is available at https://doi.org/10.7910/DVN/K2S1R2.

#### CDD training and community-based screening

Based on feedback from the pilot, we adapted existing approaches to *community-based screening*. The main changes were that CBS was implemented as a standalone activity, separate from LF MDA, and that CDDs were instructed to exclusively use a door-to-door strategy and avoid fixed-post activities. A full list of modifications on the existing approach is shown in Table 1.

**Table 1:** Existing and strengthened approach to community-based screening for lymphatic filariasis morbidity (LFM) in Côte d’Ivoire.

	Existing approach (implemented 2020)	Strengthened approach (implemented 2021)
Materials	Regional team, district team and health area supervisors provided with training of trainers document. CDDs provided with job aids, photobooks and reporting forms.	Materials reviewed and revised at workshop by NTDP team and those involved in pilot screening, including district health team and CDDs. District team and health area supervisors provided with training of trainers document. CDDs provided with job aids, photobooks and reporting forms.
Training cascade	Regional team, district team and health area supervisors trained by NTDP staff; CDDs trained by health area supervisors	District team and health area supervisors trained by NTDP staff; CDDs trained by health area supervisors with oversight from NTDP and district team.
Case-finding strategy	CDDs identify cases during MDA, using a either door-to-door or fixed-post strategy (depending on how they usually distribute medicines). Using the door to door strategy, CDDs may record cases reported by family members. Using fixed-post strategy, cases must be self-reported. CDDs encouraged to examine cases if possible but this is not mandatory.	Screening decoupled from MDA. CDDs use door-to-door strategy in all areas- no fixed-post recording. CDDs may record cases reported by family members without seeing case. CDDs encouraged to examine cases if possible but this is not mandatory.
Sensitisation	Focused primarily on MDA.	Information cascaded through district health team, regional administrative leaders, traditional leaders of the sub prefectures in the district, religious leaders, representatives of men’s, women’s and children’s groups and a communications officer. Information broadcast on district radio. Town criers informed communities about activities.
Supervision	CDD training and case identification supervised by district health team and NTDP team	CDD training and community-based screening supervised by district health team, NTDP team and/ or project team
Recording	CDDs fill patient recording forms. Patients not provided with referral ID cards.	CDDs fill patient recording forms with patient ID numbers linked to referral ID cards given to patients.

Training of CDDs was delivered through a cascade. In the first stage, health district staff and health area supervisors were trained by the head of MMDP in the NTDP (BK) using the slide deck and patient demonstrations. Health area supervisors then cascaded training to CDDs in their respective areas using the training of trainers guide, photobook and job aid, with practical demonstrations. CDDs were trained to ask to see the affected area to confirm the swelling, but were informed that they did not have to see the swelling if the person affected was unable or unwilling to show them. Following training, CDDs undertook a post-training quiz which was evaluated by the project team in order to assess the quality of the training provided.

All identified cases were provided with unique patient ID cards for re-capture during re-examination by nurses, to enable case validation. CDDs recorded cases on paper forms including patient demographic and contact details and basic clinical information and medical history. Data from these forms was entered by trained supervisors into an electronic database via electronic devices running an ODK-based application.

#### Population-based prevalence survey

We recruited health supervisors with nurse or midwife qualifications from Bongouanou district as clinical field surveyors. Twenty-six supervisors underwent a 3-day training programme on the diagnosis and management of lymphoedema and hydrocoele led by specialist and experienced dermatologists from the University Hospital of Treichville in Abidjan and by the head of the MMDP unit from the NTDP. The training included didactic material on the identification of different causes of limb swellings (including pregnancy, injury, congenital lymphedema, insect bites, allergies, and other infectious diseases) and scrotal swellings (such as hernia, congenital hydrocele, varicocele, scrotal lymphedema, tumour and haematocele). There were patient demonstrations of diagnosis of scrotal swellings and of lymphoedema, and demonstrations of limb washing for lymphoedema patients. Training materials are available in the study toolkit. Participants were assessed through a post-training test, and 18 nurses were selected for implementation.

For household (SSU) selection in PSUs, teams of 3 nurses followed separate random walks beginning from randomised start points assigned by a custom built ODK-based application. An initial household census and interview was completed to collect information on sociodemographic variables, GPS location and CDD coverage using electronic devices running an ODK-based application. Each consenting individual was checked for swelling on the limbs, and males underwent a brief testicular examination. Suspect cases were defined according to the same case definitions used by CDDs, and underwent detailed examination for confirmatory diagnosis. If any eligible participants were absent, remaining eligible household members were shown pictures of LFM from a flipbook and acted as proxy respondents. Anyone identified through this screen was defined as a suspect case and targeted for follow-up examination.

### Outcomes

The CDD case definition for suspect lymphoedema was *an increase in the volume of a limb or breast in a person aged 15 years or older* and that for suspect hydrocoele was *swollen testicles in a male aged 15 years or older*. The CDDs did not have to observe the swelling in order to record the case- they could record suspect cases reported by the person affected, or a family member in case the person was not present at the time of the visit.

The case definition of filarial lymphoedema was *swelling of limb or breast*, *in a patient aged 15 years older*, *present for at least a year but not since birth*, *and not due to leprosy*, *erysipelas*, *malignancy*, *surgery*, *or heart disease*. The definition of filarial hydrocoele was *a discrete*, *nontender mass around the testes*, *not explained by an inguinal hernia or scrotal lymphoedema*, *not present since birth and present for more than 24 hours*. Lower limb lymphoedema was classified according to the Dreyer system [24]. Cases of testicular swelling were characterised according to the system proposed by Capuano and Capuano [25].

Clinically confirmed cases of filarial lymphoedema and hydrocoele were given advice on self-care, a patient identification card, and re-imbursement for travel costs to the local health facility. Confirmed hydrocoele cases were registered for inclusion in planned hydrocoele surgery within the district, which was conducted in February 2022.

### Statistics and data analysis

We calculated the crude prevalence of suspect LFM detected by CDDs at district and health area levels using estimates of the district total and male population aged 15 years and older from the Côte d’Ivoire National Institute of Statistics as denominators [18]. Since PSU populations were not available from district health databases, design weights were assigned using population estimates extracted from the Facebook population density layer [12]. Further details are given in S1 Text. To enable spatial delineation of CDD zones, nurses walked the boundary of PSUs with CDDs, capturing the geographical limits of the catchment using GPS-enabled devices [26]. We calculated district-level prevalence estimates of LFM outcomes using *survey* package in R (version 4.1–1) [27] with post-stratification weighting applied for age and sex [18]. Prevalence estimates of LFM outcomes from community-based screening were compared to household survey prevalence estimates using risk ratios.

To understand contextual and operational factors affecting CDD coverage, we developed a mixed-effects generalized linear model (binomial distribution), for reported visitation by CDDs. We assessed sociodemographic variables at household level, including a multi-dimensional indicator of socioeconomic status (SES) constructed using latent class analysis (LCA; full details in the S2 Text) and PSU-level indicators of CDD demographics, performance and community accessibility (All candidate predictors and sources are shown in S1 Table). Continuous variables were centred and scaled. Missing data were imputed by single imputation of PSU means or modes (for continuous and categorical variables respectively). Random intercepts were allowed for CDD zones nested within health areas. Candidate variables were subject to bivariate analysis and included within final models if p < = 0.2 using likelihood ratio tests, given large parameter space and absence of observed collinearity [28]. Final models were assessed for violations of assumptions.

To estimate positive predictive value (PPV; the proportion of identified cases confirmed by gold standard diagnosis [29]) of case identification by CDDs, CDD-identified LFM cases were linked to patients examined by nurses using capture re-capture of coded patient ID cards. We estimated PPV using *epiR* [30], with confirmed diagnosis by a trained nurse as gold-standard.

We estimated the direct financial costs of CDD case finding using an ingredients-based approach to estimate costs per person targeted by the screening activity and per confirmed case identified. Costs were categorised by phase of activity (sensitisation, training of trainers (first stage of cascade), training of CDDs (second stage of cascade) and community-based screening).

### Ethics

Eligible participants (those aged 15 years and older) were provided with an information sheet and the study was explained. Written informed consent was obtained from individuals aged 18 years and above. Minors (aged <18 years) provided oral assent, and written informed consent was obtained from their parents or legal guardians. The study was granted ethical approval by Le Comité National d’Ethique des Sciences de la Vie et de la Santé in Côte d’Ivoire and the Ethics Committee of the London School of Hygiene and Tropical Medicine (Reference 21203).

## Results

### Study participants

Across 110 PSUs, nurses visited 8,247 households which were occupied and had an adult present. Of these, 58 (0.70%) refused to participate. Across 8,189 households, 12,289 people were invited to participate and 12,287 (99.99%) agreed, including 4,818 males (39.2%). Twenty participants (0.16%) refused limb examinations and 202 males (4.2%) refused scrotal examination. The median number of households visited per cluster was 75 (interquartile range [IQR] 61–86), and the median number examined per cluster was 98 (IQR 77–112).

### Reliability of community-based screening

**Fig 1:**
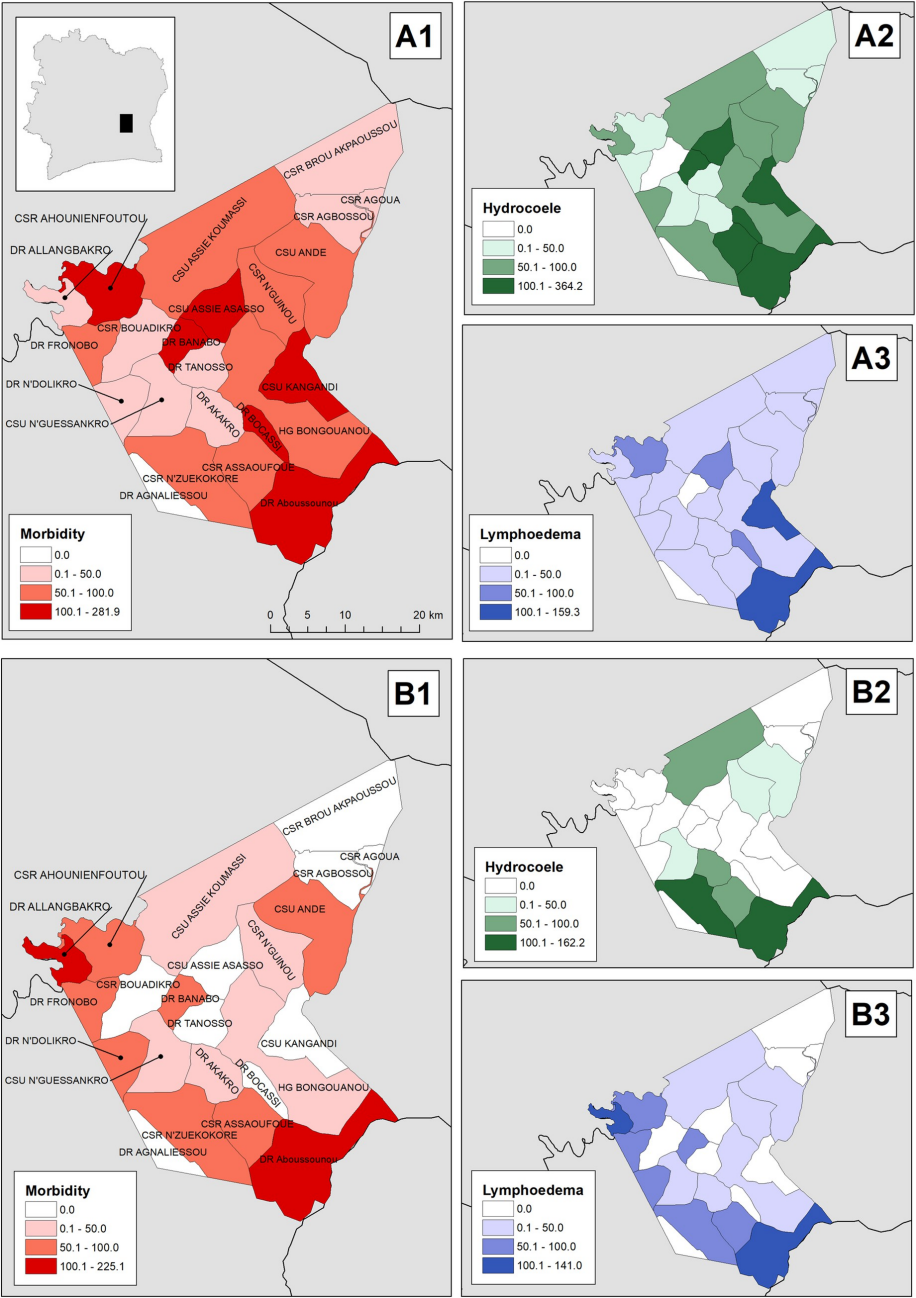
Prevalence (number of cases per 10,000 population) of 1) lymphatic filariasis morbidity, 2) filarial lymphoedema, and 3) hydrocoele, detected through A) community-based screening led by volunteers and B) population-based prevalence survey led by formally trained nurses in Bongouanou, Côte d’Ivoire. Base-map contains district boundaries from the United Nations Office for the Coordination of Humanitarian Affairs (OCHA) {Affairs, 2019 #164}.

Following an exhaustive case search across the district among people > = 15 years old (population 94,319; 47,349 males), CDDs identified 824 suspect LFM cases: 374 lymphoedema, 457 hydrocoele, and 7 with both (Table 2). The prevalence of suspect LFM was 87.4 per 10,000 (95% CI 81.5–93.5 per 10,000). The prevalence of suspect lymphoedema was 39.7 per 10,000 (95% CI 35.7–43.9 per 10,000) and that of suspect hydrocoele96.5 per 10,000 males (95% CI 87.9–105.7 per 10,000) (Fig 1).

**Table 2:** Number and prevalence (per 10,000) of LFM cases detected through community-based screening and population-based survey.

	Community-based screening				Re-examined by nurses			Population-based survey^1^					*Prev*. *ratio (screen*:*survey)*
	Screened (N)	Suspect cases (n)	Crude prev.	*(95% CI)*	N	Con-firmed	PPV	Examined (N)	confirmed cases (n)	Weighted			
										Estimated cases	Prev.	*(95% CI)*	
LF morbidity	94,319	824	87.4	*(81*.*5–93*.*5)*	315	150	47.1	12,267	53	475	50.3	*(43*.*4–57*.*8)*	*1*.*73 (1*.*55–1*.*94)*
Al leg swellings	94,319	374	39.7	*(35*.*7–43*.*9)*	120	93	77.5	12,267	49	507	53.8	*(44*.*6–63*.*7)*	*0*.*74 (0*.*65–0*.*84)*
LF lymphedema						78	65		36	332	35.2	*(30*.*3–40*.*5)*	*1*.*13 (0*.*97–1*.*31)*
All scrotum swellings	47,349	457	96.5	*(87*.*9–105*.*7)*	191	179	93.7	4,613	50	432	91.3	*(83*.*2–99*.*8)*	*1*.*06 (0*.*93–1*.*21)*
LF hydrocele						65	34		18	156	33.0	*(27*.*7–38*.*8)*	*2*.*93 (2*.*46–3*.*55)*

^1^ Prevalence estimates adjusted for survey design and post-stratified on age group and gender. PPV = predicted value positive. Prev. = prevalence.

In the population-based survey, 53 cases of LFM were confirmed, giving a prevalence of 50.3 per 10,000 (95% CI 43.4–57.8) after adjusting for survey design and non-response. The prevalence ratio of CDD-identified suspect LFM to confirmed LFM was 1.73 (95% CI 1.55–1.94). Forty-nine cases of leg swellings were identified and 36 were confirmed as filarial lymphoedema. The design-adjusted prevalence estimate of confirmed filarial lymphoedema was 35.2 (95% CI 30.3–40.5) per 10,000; within confidence bounds of the CDD estimate (prevalence ratio of 1.13 [95% CI 0.97–1.31]). Fifty cases of scrotal swellings were identified and 18 confirmed as filarial hydrocoele. The adjusted prevalence of scrotal swellings was 91.3 per 10,000 males (95% CI 83.2–99.8). The prevalence ratio for scrotal swellings was 1.06 (95% CI 0.93–1.21). The prevalence of confirmed filarial hydrocoele was 33.0 per 10,000 (95% CI 27.7–38.8), with a prevalence ratio of 2.93 (95% CI 2.46–3.55). Two cases presented both filarial lymphoedema and hydrocoele.

### Cases missed by CDDs

Of 36 confirmed filarial lymphoedema cases identified in the population-based prevalence survey, 11 (30.6%) had not been identified despite the household having been visited. Of 50 cases of scrotal swellings identified in the population-based survey, 15 (30.0%) had been missed despite their household being visited. The low number of missed cases limited precise comparisons between those missed and identified.

### Reliability of LFM case identification by CDDs

To assess reliability of case identification, nurses examined cases identified by CDDs at post-survey clinics. Of 374 suspect lymphoedema cases, 120 (32.1%) were re-examined and leg swellings were confirmed in 93 cases, giving a PPV of 77.5% (96% CI 69.0–84.6%). Filarial lymphoedema was confirmed in 81 of the 120 cases (PPV 65.0%, 95% CI 55.8–73.5%). The 15 cases with leg swelling not diagnosed as filarial lymphoedema included 4 cases of Buruli ulcer, 3 of erysipelas, and 9 other diagnoses.

Of 457 suspect hydrocoele cases identified by CDDs, 191 (41.8%) underwent scrotal examination by nurses. Scrotal swelling was confirmed in 179. The PPV for identification of scrotal swellings was 93.7% (95% CI 89.3–96.7%). Filarial hydrocoele was confirmed in 65 cases (PPV 34.0%, 95% CI 27.3–41.2%). Among the 126 non-filarial scrotal swellings, 103 were cases of hernia, 8 were congenital hydrocele, and 3 were other diagnoses. It is important to note that CDDs were not asked to differentiate between filarial and non-filarial aetiology for suspected case definitions (see Methods).

### Household coverage and equity of community-based screening

Of 8,189 households interviewed by nurses, 5,265 (64.3%; 95% CI 63.2–65.3%) reported being visited by a CDD during CBS; 2,769 (33.8%) had not; and 154 (1.9%) did not know. Using causal modelling approaches to account for household, PSU and health-area contextual factors, household size was positively associated with inclusion in the CBS (Table 3). Households in which the primary language of the household head was Baoule, Senoufo, Dioula or other languages were less likely to have been visited than those in the predominant ethno-lingual group of Bongouanou, Agni. Households owning a basic mobile or smartphone were also more likely to have been visited than those without, independent of socioeconomic status. Crudely, households in the lowest socioeconomic class based on LCA were more likely to have been visited than those in the middle and highest classes, but after adjusting for survey design and other covariates, the middle SES class was more likely to have been visited. PSU-level fixed effects suggested higher coverage in more socioeconomically-developed clusters, with a positive association of household coverage with stable night light. A very low proportion of variance was attributable to health area levels (ICC L1 = 0.099) indicating little effect of implementation at this scale.

**Table 3:** Predictors of household inclusion in community case search.

Predictors of	n visited by CDD	N in PBPS*	Risk (%)	Multivariate Analysis	
visitation by CDDs				Adjusted OR (95% CI)	LRT p-value
**Household-level Fixed Effects**	** **	** **	** **	** **	** **
**Log household size**			* *	1.07 (1.06–1.09)	<0.001
**Phone ownership** ^**1**^			* *		<0.001
No phone	747	1,303	*57*.*33*	1 (base)	
Basic phone only	740	1,156	*64*.*01*	1.11 (1.07–1.15)	
Smartphone	3,645	5,419	*67*.*26*	1.06 (1.03–1.09)	
**Language** ^**2**^			* *		<0.001
Agni	3,998	6,022	*66*.*39*	1 (base)	
Baoule	322	511	*63*.*01*	0.94 (0.91–0.98)	
Dan	10	21	*47*.*62*	0.94 (0.78–1.12)	
Dioula	287	492	*58*.*33*	0.93 (0.90–0.97)	
French	18	33	*54*.*55*	1.18 (1.01–1.38)	
Malinke	110	160	*68*.*75*	0.96 (0.89–1.02)	
Senoufo	33	59	*55*.*93*	0.88 (0.79–0.98)	
Other/ Unknown	354	580	*61*.*03*	0.92 (0.89–0.96)	
**Socioeconomic status** ^**3**^			* *		0.001
Lowest	504	658	*76*.*6*	1 (base)	
Middle	2,925	4,284	*68*.*28*	1.06 (1.01–1.10)	
Highest	1,703	2,936	*58*	1.02 (0.98–1.07)	
**Cluster/CDD-level Fixed Effects**	** **	** **	** **	** **	** **
**CDD score** ^**4**^			* *	1.04 (1.00–1.08)	0.058
**CDD gender**			* *		0.083
Female	1,722	2,474	*69*.*6*	1 (base)	
Male	3,410	5,404	*63*.*1*	0.93 (0.86–1.01)	
**Mean distance to stable lights**			* *	1.06 (1.00–1.12)	0.047
**Mean accessibility to HFs**			* *	1.03 (0.99–1.07)	0.123
** **	** **	** **	** **	**ICC**	** **
				CDD zones within health areas (L1:L2)	0.099
				CDD zones (L2)	0.145

*PBPS = population-based prevalence survey.

^1^ Missing for 1 household.

^2^ Missing for 9 households.

^3^ Multidimensional indicator constructed from: household electricity connection (missing for 5 households), education of household head (missing for 1 household), dwelling walls made from improved material (missing for 3 households), dwelling floor made from improved material (missing for 1 household), household access to improved water supply (missing for 3 households). Households missing data were assigned the cluster modal value.

^4^ Missing for 3 CDDs equating to 156 households, which were not included in this model.

### Patient clinical characteristics

Of the 125 confirmed cases of filarial lymphoedema, the majority (58.4%) were female and the median age was 48.0 years (interquartile range; IQR 32.4–57.0) (S2 Table). Most (60.8%) had unilateral lower limb swelling and the median duration of swelling was 7.0 years. Approximately a third (29.6%) had never experienced an acute attack, while 47.2% experienced less than one per month and 20.8% experienced at least one per month. Two thirds (66.7%) of cases were Dreyer stage one or two, and around half (47.2%) had entry lesions at the time of examination.

The median age of the 107 confirmed filarial hydrocoele cases was 52.0 years (IQR 40.5–64.0), and the median duration of swelling was 5.0 years (IQR 3.0–10.0). Around half (54.4%) said they never experienced acute attacks, 21.6% experienced at least one per month and 12.0% experienced at least one per month. By the staging and grading system proposed by Capuano and Capuano [25], most cases (72.1%) were classified beyond stage 2 (scrotum larger than a tennis ball), but few (10.4%) were beyond grade 1 (visible burial of the penis). Patient characteristics are summarised in the S2 Table.

### Financial costs

The overall direct financial cost of CBS was 26,678.36 USD, of which approximately half was spent on preparation (sensitisation, training of trainers and of CDDs) and half on door-to-door screening by CDDs (S2 Fig). The cost per suspect case identified by CDDs was $33.60, that per case confirmed was $69.62 and that per person targeted was $0.17.

## Discussion

In this study, we evaluated a community-based strategy to estimate LFM burden, which can be implemented as a scalable, programmatic activity to support elimination of LF as a public health problem [7]. The strategy was programmatically feasible in terms of cost per person examined, and provided comparable prevalence estimates relative to a rigorous population-based prevalence survey. These were 40 times higher than those obtained in recent pilots implemented during MDA in Côte d’Ivoire. Taken together, these results indicate that the strengthened, standalone strategy appears a considerably more effective approach to describe the true epidemiological situation of LFM. We also quantified the coverage and equity of our community-based strategy, and identified challenges faced by CDDs in differentiating hydrocoele from scrotal swellings of other causes. Whether this latter challenge can be overcome cost-effectively remains an open question for the GPELF and LF-endemic countries.

We made a series of simple changes to the existing programmatic strategy that was piloted during MDA in 2020 in Côte d’Ivoire. The modifications included strengthening of the training programme, increased supervision, and de-coupling of case-finding from MDA. These appear to have facilitated a step-change improvement in LFM case enumeration, resulting in prevalence estimates 40 times higher than those from initial pilots [19]. All modifications are likely to have contributed to improved reliability of case detection, though further research, including process evaluation at scale, would be needed to elucidate the contribution of each element to success at different levels of implementation. We believe the de-coupling of case-finding from MDA was a significant enabler. Previous studies have shown dedicated case searches to be more effective than those embedded in other activities, suggesting that competing demands, rather than de-centralisation *per-se*, are a more important barrier to reliable community-led LFM estimation [16, 17].

Although the estimate of LFM prevalence detected by CDDs was higher than that shown by the population-based prevalence survey, we consider the CDD-estimate reflective of the epidemiological situation in Bongouanou. CDDs appeared to face different challenges in the quantitation of suspect lymphedema and suspect hydrocele. Their identification of lymphoedema was good, though imperfect, in terms of both reliability and sensitivity: the PPV of 65% indicates that around one third of suspect cases identified by CDDs were not due to filarial lymphoedema, while the survey suggested that CDDs missed around a third of true cases (11 of 36 confirmed filarial lymphoedema cases had not been identified in the CBS). In effect, the similar magnitudes of these parameters resulted in an estimate of suspect LF-lymphoedema prevalence close to (within the confidence range of) that found in the population-based prevalence survey. This is an encouraging result in terms of obtaining accurate estimates of lymphoedema prevalence through CBS, though the sensitivity of case identification could be further improved through modifications to the CDD training materials or community education to raise awareness of early signs of lymphoedema.

The estimate of scrotal swelling prevalence by CDDs was close to (within confidence bounds of) that from the population-based prevalence survey, and the PPV of case identification for scrotal swellings was 93.7% (indicating that fewer than 7% of suspect cases were misdiagnosed). This suggests that CBS is both sensitive and specific as a tool for enumeration of scrotal swellings. It is important to emphasise that CDDs could record cases without physical examination while nurses could not confirm cases without examination. Among the 202 men who did not undergo scrotal examination during the population-based prevalence survey, there may have been cases who were willing to describe symptoms to a CDD but not to be examined by a nurse in the household setting, and thus went undetected.

The low PPV of hydrocoele identification led to an overestimation of hydrocoele prevalence by CDDs. This was not unanticipated, since CDDs were not trained to distinguish the aetiology of scrotal swellings, which was deemed unfeasible, particularly given the scale of coverage expected of them. A similarly low PPV of hydrocoele identification by CDDs has been demonstrated in Ghana, though the same study demonstrated a much higher PPV (92%) in Malawi [16]. Differentiation of hernia from hydrocoele may be possible with training [16], but given resource limitations within the GPELF, this may be unrealistic at scale. As this activity is scaled-up, suspect hydrocoele cases would need to be confirmed by a local health worker or a pre-surgical team prior to corrective surgery. Re-examination of suspect cases would enable estimation of local PPVs, which could be used to adjust district-level estimates of hydrocoele prevalence generated by CBS. However, under current funding structures, cases are only eligible for free surgical intervention if confirmed as filarial hydrocoele, despite the fact that the repair of groin hernia, by far the most prevalent alternative diagnosis in our study, is also resolved by a simple and cost-effective surgery [31]. Our findings reinforce the clear public health and economic arguments for integration of case finding and surgery for these conditions, which should receive consideration within LF endemic countries [32]. The reliability of lymphoedema identification by CDDs was much higher, with PPV similar to estimates from other settings [16].

An important consideration for our findings is the potential scalability of the approach. This is crucial as WHO elimination dossiers for LF necessitate exhaustive enumeration of LFM across all endemic and previously endemic areas, often covering very large populations. We believe the financial cost per person screened in our study was low from an NTD programmatic perspective, being comparable to the cost per person treated through MDA in African settings in the early 2000’s [33], and substantially lower than the cost per person examined in the global trachoma mapping project ($4.20 in Côte d’Ivoire in 2015) [34]. The cost per case confirmed was lower than published estimates of the cost per case found in community-based leprosy screening, varying from $72 (in Mali, 1999)- $313 (in Nigeria, 2002) [35]. The costs were also low relative to the financial burden on patients unable to work due to LFM, estimated at around $700 per individual affected [36], and costing almost $1.3 billion a year in lost productivity globally [37]. Hydrocoele surgeries are extremely cost-effective [38], and lymphoedema management costs far less than the economic benefits to patients over their lifetime [39]. Taken together, these results present a strong case for investment in community-led approaches to identify LFM cases to be linked to MMDP services. Parallel investment in universal health coverage and surveillance strengthening will be required for the sustainability of these programmes.

The level of household coverage achieved by CDDs was high, aligning to WHO coverage targets for LF MDA [40]. However, we identified factors at household and community (CDD zone) level that affected the probability of household inclusion. Within communities, households in the middle socioeconomic class were more likely to have been visited, while some minority language groups appeared at risk of exclusion. This may reflect CDD bias towards friends or those of specific social standing, which has been demonstrated in the context of MDA in Uganda [41]. To address this, the inclusion and sensitisation of minority language groups should be planned from early stages, and communications materials may need to be translated into multiple languages before implementation. There was a low level of variability at health area level, which supports the quality of the training cascade, given that CDDs were trained through supervisors at health area level.

Our study had several limitations. Both case-finding methods were fallible to ascertainment bias, as males, students, and people of working age would be less likely to be at home. Although we adjusted the survey prevalence estimate to correct this bias, it was not possible to do the same for the community-based estimate. Further, people affected by LFM may have been more likely to be at home- either because of unemployment or because they stayed intentionally for the survey. Whilst our population-based prevalence survey was conducted following specialist clinical training, outcome measures were based on clinical diagnosis made by non-physician healthcare workers, which may be imperfect. Selection bias may have been introduced by random walk procedures. While we aimed to nullify this by using multiple, random start points, random walk is more prone to selection bias than fully randomised or segment-based sampling [42]. Another limitation is that not all suspect cases identified by CDDs presented for re-examination by nurses. This may have resulted in biased estimates of PPV if true LFM cases (potentially those with more severe symptoms) were more likely than false positives to present.

We have demonstrated the effectiveness of a scalable, community-based case-finding approach to LFM burden estimation. Together with our findings, the extensive toolkit we present can support programmes planning to implement similar activities. We believe this study transparently demonstrates how community-based infrastructure can support LF elimination, and is generalisable to other LF-endemic settings. Important advocacy points raised by our findings include the potential benefits of de-coupling LFM case-finding from MDA and the overt operational and public health benefits of integrating the detection and management of hydrocoele with scrotal hernia.

## Supporting information

S1 Checklist STROBE statement—checklist of items that should be included in reports of cross-sectional studies. (DOCX)

S1 File (DOCX)

S1 Fig Map of the study area. Population density data is from the Worldpop project: Linard C, Gilbert M, Snow RW, Noor AM, Tatem AJ. Population distribution, settlement patterns and accessibility across Africa in 2010. PloS one. 2012;7(2):e31743, www.worldpop.org [accessed 03/10/2020]. Base-map contains district and subdistrict boundaries from United Nations Office for the Coordination of Humanitarian Affairs (OCHA): (Côte d’Ivoire—Subnational Administrative Boundaries. 2019.), accessed 16/01/2022, roads from OpenStreetMap: HOTOSM Côte d’Ivoire Roads (OpenStreetMap Export), accessed via the Humanitarian Data Exchange website, and georeferenced health facility locations: Maina J, Ouma PO, Macharia PM, Alegana VA, Mitto B, Fall IS, et al. A spatial database of health facilities managed by the public health sector in sub Saharan Africa. Scientific data. 2019;6(1):1–8.
(TIF)

S2 Fig Financial costs of CDD screening activity, per person targeted and by case confirmed (total and by phase). (PNG)

S1 Text Estimation of populations within survey strata and clusters. (DOCX)

S2 Text Constructing multidimensional indicator of socioeconomic status (SES) using latent class analysis (LCA). (DOCX)

S1 Table Candidate predictors and sources assessed for inclusion in mixed-effects generalized linear model of inclusion in household screening. (DOCX)

S2 Table Demographic and clinical characteristics of confirmed cases of lymphatic filariasis morbidity identified. (DOCX)
