# Supplementary material for: Effectiveness of community-based burden estimation to achieve elimination of lymphatic filariasis: A comparative cross-sectional investigation in Côte d’Ivoire
Source: PLOS Glob Public Health. 2022 Aug 31;2(8):e0000760. doi: 10.1371/journal.pgph.0000760 (PMC10022321; doi:10.1371/journal.pgph.0000760)
Supplement: S1 File — (DOCX) [file pgph.0000760.s002.docx]

**Abstract**

For lymphatic filariasis (LF) elimination, endemic countries must document the burden of LF morbidity (LFM). Community-based screening (CBS) is used to collect morbidity data, but evidence demonstrating its reliability is limited. Recent pilots of CBS for LFM alongside mass drug administration (MDA) in Côte d’Ivoire suggested low LFM prevalence (2.1-2.2 per 10,000).

We estimated LFM prevalence in Bongouanou District, Côte d’Ivoire, using a comparative cross-sectional design. We compared CBS implemented independently of MDA, adapted from existing Ministry of Health protocols, to a population-based prevalence survey led by formally trained nurses. We evaluated the reliability of case identification, coverage, equity, and cost of CBS.

CBS identified 87.4 cases of LFM per 10,000; the survey identified 47.5 (39.4-56.3; prevalence ratio [PR] 1.84; 95% CI 1.64-2.07). CBS identified 39.7 cases of suspect lymphoedema per 10,000; the survey confirmed 35.1 (29.2-41.5) filarial lymphoedema cases per 10,000 (PR 1.13 [0.98-1.31]). CBS identified 100.3 scrotal swellings per 10,000; the survey found 61.5 (55.5-67.8; PR 1.63 [1.41-1.88]); including 26.6 (21.5-32.4) filarial hydrocoele per 10,000 (PR of suspect to confirmed hydrocele 3.77 [3.12-4.64]). Positive predictive values for case identification through CBS were 64.0% (54.5-72.8%) for filarial lymphoedema; 93.2% (88.5-96.4%) for scrotal swellings; and 33.3% (26.4-40.8%) for filarial hydrocoele. Households of lower socioeconomic status and certain minority languages were at risk of exclusion. Direct financial costs were $0.17 per individual targeted and $69.62 per case confirmed. We provide our CBS toolkit.

Our community-based approach to LFM burden estimation appears scalable and provided reliable prevalence estimates for LFM, scrotal swellings and LF-lymphoedema. The results represent a step-change improvement on CBS integrated with MDA, whilst remaining at programmatically feasible costs. Filarial hydrocoele cases were overestimated, attributable to the use of case definitions suitable for mass-screening by informal staff. Our findings are broadly applicable to countries aiming for LF elimination using CBS.

**Résumé**

Pour l'élimination de la filariose lymphatique (FL), les pays endémiques doivent documenter le fardeau de la morbidité due à la FL (LFM). Le dépistage communautaire (CBS) est utilisé pour recueillir des données sur la morbidité, mais les preuves démontrant sa fiabilité sont limitées. Des enquêtes pilotes récentes de dépistage communautaire pour la morbidité due à la FL parallèlement à l'administration massive de médicaments (MDA) en Côte d'Ivoire ont montré une faible prévalence de LFM (2,1-2,2 pour 10 000).

Le dépistage communautaire a identifié 87,4 cas de LFM pour 10 000 ; l'enquête a identifié 47,5 (39,4-56,3 ; ratio de prévalence [RP] 1,84 ; IC à 95% 1,64-2,07). Le dépistage communautaire a identifié 39,7 cas de lymphœdème suspect pour 10 000 ; l'enquête a confirmé 35,1 (29,2-41,5) cas de lymphœdème filarien pour 10 000 (PR 1,13 [0,98-1,31]). Le dépistage communautaire a identifié 100,3 gonflements scrotaux pour 10 000 ; l'enquête a trouvé 61,5 (55,5-67,8 ; PR 1,63 [1,41-1,88]) ; dont 26,6 (21,5-32,4) hydrocèles filaires pour 10 000 (PR de l'hydrocèle suspectée à l'hydrocèle confirmée 3,77 [3,12-4,64]). Les valeurs prédictives positives pour l'identification des cas par le dépistage communautaire étaient de 64,0 % (54,5-72,8 %) pour le lymphœdème filarien, de 93,2 % (88,5-96,4 %) pour les gonflements scrotaux et de 33,3 % (26,4-40,8 %) pour l'hydrocèle filarienne. Les ménages de statut socio-économique inférieur et certaines langues minoritaires étaient à risque d'exclusion. Les coûts financiers directs étaient de 0,17 $ par individu ciblé et de 69,62 $ par cas confirmé. Nous fournissons notre boîte à outils pour le dépistage communautaire.

Notre approche communautaire de l'estimation de la charge de la FLM (morbidité due à la FL) semble extensible et a fourni des estimations fiables de la prévalence de la FLM, des gonflements scrotaux et des lymphœdèmes. Les résultats représentent un changement radical par rapport au dépistage communautaire intégrée à la MDA, tout en restant à des coûts programmables. Les cas d'hydrocèle filarienne ont été surestimés, ce qui est attribuable à l'utilisation de définitions de cas adaptées au dépistage de masse par du personnel non formé. Nos résultats sont largement applicables aux pays visant l'élimination de la FL à l'aide du dépistage communautaire.
