# Supplementary figures and images for: Effectiveness of community-based burden estimation to achieve elimination of lymphatic filariasis: A comparative cross-sectional investigation in Côte d’Ivoire
Source: PLOS Glob Public Health. 2022 Aug 31;2(8):e0000760. doi: 10.1371/journal.pgph.0000760 (PMC10022321; doi:10.1371/journal.pgph.0000760)

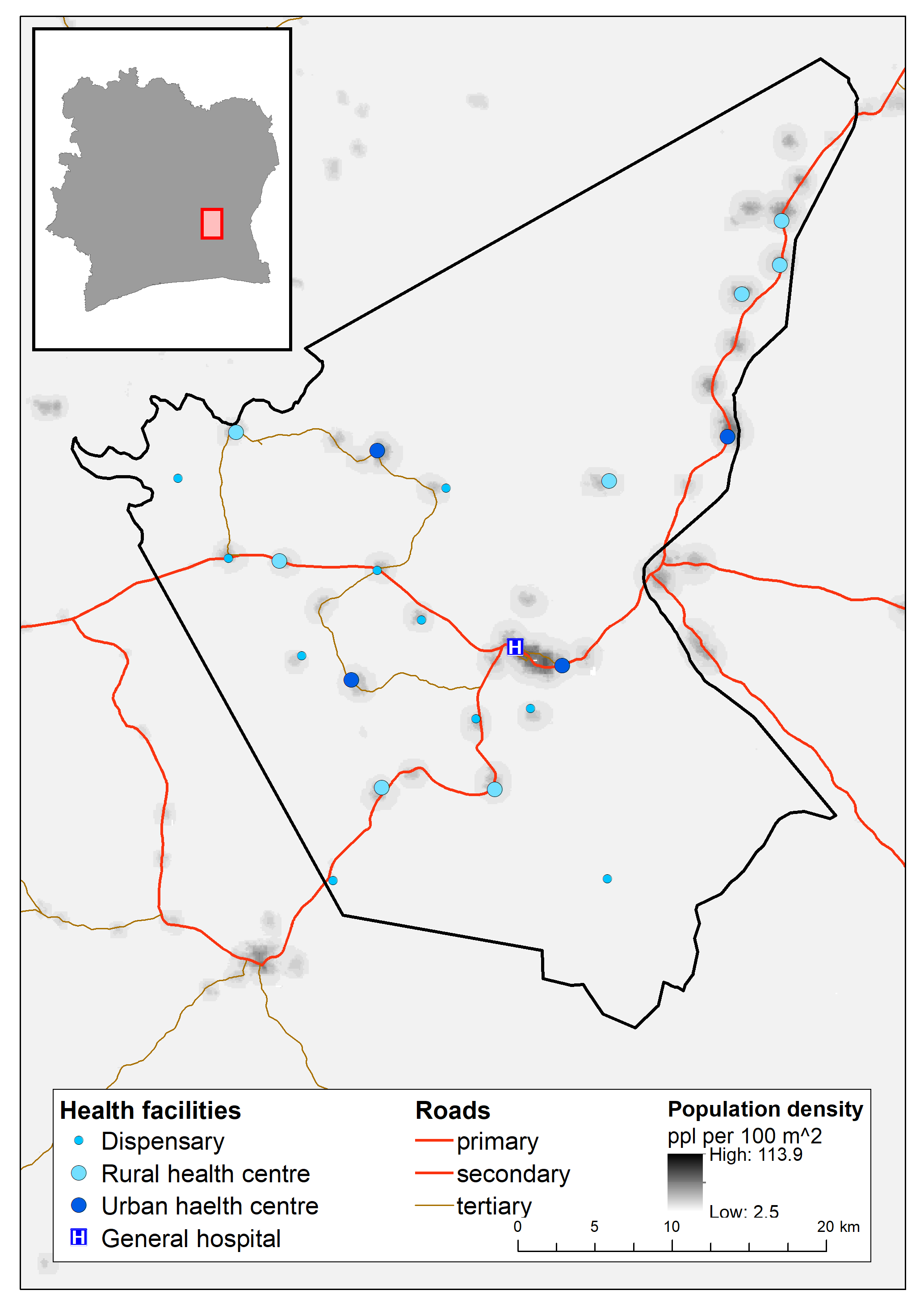

Supplement: S1 Fig — Population density data is from the Worldpop project: Linard C, Gilbert M, Snow RW, Noor AM, Tatem AJ. Population distribution, settlement patterns and accessibility across Africa in 2010. PloS one. 2012;7(2):e31743, www.worldpop.org [accessed 03/10/2020]. Base-map contains district and subdistrict boundaries from United Nations Office for the Coordination of Humanitarian Affairs (OCHA): (Côte d’Ivoire—Subnational Administrative Boundaries. 2019.), accessed 16/01/2022, roads from OpenStreetMap: HOTOSM Côte d’Ivoire Roads (OpenStreetMap Export), accessed via the Humanitarian Data Exchange website, and georeferenced health facility locations: Maina J, Ouma PO, Macharia PM, Alegana VA, Mitto B, Fall IS, et al. A spatial database of health facilities managed by the public health sector in sub Saharan Africa. Scientific data. 2019;6(1):1–8. (TIF) [file pgph.0000760.s003.tif]

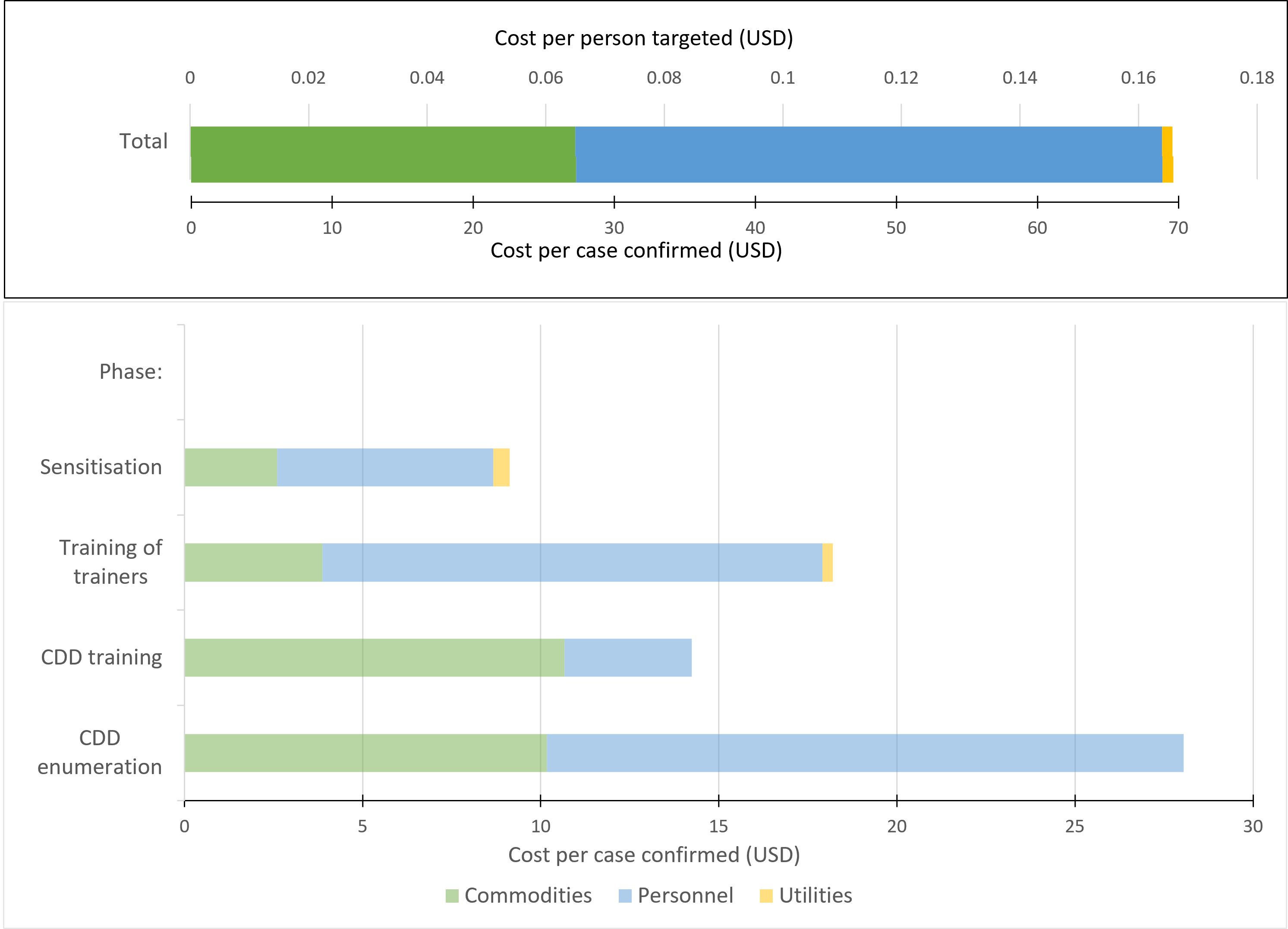

Supplement: S2 Fig — (PNG) [file pgph.0000760.s004.png]
