# Supplementary material for: Effectiveness of community-based burden estimation to achieve elimination of lymphatic filariasis: A comparative cross-sectional investigation in Côte d’Ivoire
Source: PLOS Glob Public Health. 2022 Aug 31;2(8):e0000760. doi: 10.1371/journal.pgph.0000760 (PMC10022321; doi:10.1371/journal.pgph.0000760)
Supplement: S1 Text — (DOCX) [file pgph.0000760.s005.docx]

**S1 Text: Supplementary Methods: Estimation of populations within survey strata and clusters**

Health area boundaries were mapped based catchment areas, generated using an accessibility surface representing estimated travel time to the nearest health facility. We used the Cost Allocation tool in ArcMap to determine the most accessible health facility from each point in the district, using the precise locations of health facilities as the feature source data and the travel time surface as the input cost raster. We converted the output raster to a polygon shapefile. After the field survey, the estimated health area boundaries were manually adjusted to ensure each area encompassed the boundaries of mapped clusters within it.

We extracted the total population within mapped health area boundaries from the population density layer produced by the Facebook Connectivity Lab [1]. We compared the extracted population estimate to the reported population estimate used for operational purposes within the district and calculated a “correction factor” to re-scale health area populations to the reported populations.

Cluster boundaries were mapped by field teams accompanied by the CDD responsible for the area. Field teams recorded the limits of the zone through a “geotrace” form running from SurveyCTO software on Samsung smartphones. This automatically captured the GPS location every 20 seconds or whenever the data collector manually recorded the location. The field team were trained to pass behind houses on the boundary of the zone where possible, and to check with the CDD if any paths leading away from the main road led to houses within the zone, to ensure the full extent of the zone was captured.

Total and male populations were extracted from the mapped cluster boundaries and re-scaled by the correction factor to align the estimate to the reported population. The population estimates were multiplied by the fraction of the population aged 15 years and older in Bongouanou district [2].

**References**

1. Facebook Connectivity Lab and Center for International Earth Science Information Network - CIESIN - Columbia University. High Resolution Settlement Layer (HRSL). Source imagery for HRSL ©. 2016 DigitalGlobe. Accessed 10/03/2020.

2. Institut National de la Statistique (INS) UNFPA. Côte d'Ivoire population statistics. In: ROWCA O, editor. 2019.
