# Supplementary material for: Effectiveness of community-based burden estimation to achieve elimination of lymphatic filariasis: A comparative cross-sectional investigation in Côte d’Ivoire
Source: PLOS Glob Public Health. 2022 Aug 31;2(8):e0000760. doi: 10.1371/journal.pgph.0000760 (PMC10022321; doi:10.1371/journal.pgph.0000760)
Supplement: S2 Text — (DOCX) [file pgph.0000760.s006.docx]

**S2 Text: Supplementary Methods: Constructing multidimensional indicator of socioeconomic status (SES) using latent class analysis (LCA)**

We used the LCAvarsel package in R [1] to identify the most appropriate predictors and number of classes for LCA using the Fop et al. 2017 method which is described as a “greedy” backwards/forwards selection procedure, starting with all candidate predictors. The algorithm iteratively removes variables, tests the change in Bayesian Information Criterion (BIC) to determine whether to accept or reject the removal, adds each previously removed variable back in after successive removals and re-testing the change in BIC to determine whether to accept or reject re-addition.

The variables selected were: household electricity connection, education of household head, dwelling walls made from improved material, dwelling floor made from improved material, household access to improved water supply.

**References**

1. Fop MaM, T. B. LCAvarsel: Variable selection for latent class analysis. R package version 1.1. 2017.
