# Supplementary material for: Effectiveness of community-based burden estimation to achieve elimination of lymphatic filariasis: A comparative cross-sectional investigation in Côte d’Ivoire
Source: PLOS Glob Public Health. 2022 Aug 31;2(8):e0000760. doi: 10.1371/journal.pgph.0000760 (PMC10022321; doi:10.1371/journal.pgph.0000760)
Supplement: S1 Table — (DOCX) [file pgph.0000760.s007.docx]

**S1 Table: Candidate predictors and sources assessed for inclusion in mixed-effects generalized linear model of inclusion in household screening.**

| **Level** | **Variable** | **Source** | **Selected** |
| --- | --- | --- | --- |
| Household | Log household size (scaled) | Household-level questionnaire | 1 |
| Household | Socioeconomic status (low, middle, high) | Multi-dimensional indicator generated from a combination of questions from household-level questionnaire | 1 |
| Household | Phone ownership | Household-level questionnaire | 1 |
| Household | Primary language of household head | Household-level questionnaire | 1 |
| Household | Household within a campment | Household-level questionnaire | 0 |
| Household | Any children in household | Household-level questionnaire | 0 |
| Household | Number of suspect cases in household | Household-level questionnaire | 0 |
| Cluster (CDD) | Primary language of CDD | CDD pre-post quiz | 0 |
| Cluster (CDD) | CDD age | CDD pre-post quiz | 0 |
| Cluster (CDD) | CDD gender | CDD pre-post quiz | 1 |
| Cluster (CDD) | CDD score on training quiz | CDD pre-post quiz | 1 |
| Cluster (zone) | Mean travel time to nearest health facility | Mean travel time extracted from pixels within geo-traced cluster boundary [1] | 1 |
| Cluster (zone) | Mean Euclidean distance to stable night lights | Mean Euclidean distance from pixels within geo-traced cluster boundary to the nearest stable light from National Oceanic and Atmospheric Administration (NOAA) [2] | 1 |
| Cluster (zone) | Urban-rural classification | Modal category (rural, urban, peri-urban) from Center for International Earth Science Information Network [3,4] within geo-traced cluster boundary | 0 |
| Cluster (zone) | Log CDD zone area (scaled) | Calculated from geo-traced cluster boundary | 0 |
| Cluster (zone) | Log CDD zone population (scaled) | Total population extracted from Facebook Connectivity Lab population density surface [5] within geo-traced cluster boundary | 0 |

**References**

1. Mary Hahm et al. Spatial analysis of accessibility to health facilities for lymphatic filariasis patients in Cote d'Ivoire. In prep.

2. (NOAA) NOaAA. DMSP-OLS Nighttime Lights Time Series. Boulder, CO: National Geophysical Data Center. 4 ed2014.

3. Center for International Earth Science Information Network - CIESIN - Columbia University, International Food Policy Research Institute - IFPRI, The World Bank, Centro Internacional de Agricultura Tropical - CIAT. Global Rural-Urban Mapping Project, Version 1 (GRUMPv1): Urban Extents Grid. Palisades, NY: NASA Socioeconomic Data and Applications Center (SEDAC); 2011.

4. Balk DL, Deichmann U, Yetman G, Pozzi F, Hay SI, Nelson A. Determining Global Population Distribution: Methods, Applications and Data. Advances in Parasitology. 2006;62:119-56.

5. Facebook Connectivity Lab and Center for International Earth Science Information Network - CIESIN - Columbia University. High Resolution Settlement Layer (HRSL). Source imagery for HRSL ©. 2016 DigitalGlobe. Accessed 10/03/2020.
