# Supplementary material for: Effectiveness of community-based burden estimation to achieve elimination of lymphatic filariasis: A comparative cross-sectional investigation in Côte d’Ivoire
Source: PLOS Glob Public Health. 2022 Aug 31;2(8):e0000760. doi: 10.1371/journal.pgph.0000760 (PMC10022321; doi:10.1371/journal.pgph.0000760)
Supplement: S2 Table — (DOCX) [file pgph.0000760.s008.docx]

**Table 2S: Demographic and clinical characteristics of confirmed cases of lymphatic filariasis morbidity identified**

| **Demographic/ clinical characteristics** | | **Confirmed filarial lymphedema (n=125)** | | | **Demographic/ clinical characteristics** | | **Confirmed filarial hydrocele (n=111)** | | |
| --- | --- | --- | --- | --- | --- | --- | --- | --- | --- |
|  |  | **N** | ***% / median*** | ***CI/ IQR*** |  |  | **N** | ***% / median*** | ***CI/ IQR*** |
| **Gender** | |  |  |  |  |  |  |  |  |
|  | Female | 73 | *58.4* | *49.2- 67.1%* |  |  |  |  |  |
|  | Male | 52 | *41.6* | *32.4- 50.6 %* |  |  |  |  |  |
| **Age (median)** | |  | *48* | *35.0- 57.0* | **Age (median)** | |  | *52* | *40.5- 64.0* |
| **Years of swelling (median)** | |  | *7* | *3.0- 15.0* | **Years of swelling (median)** | |  | *5* | *3.0-10.0* |
| **Location of swelling** | |  |  |  | **Condition scrotal skin** | |  |  |  |
|  | Upper limb, unilateral | 3 | *2.4* | *0.5- 6.9%* |  | Normal | 76 | *60.8* | *59.0- 77.0%* |
|  | Lower limb, unilateral | 76 | *60.8* | *51.7- 69.4%* |  | Thickened | 35 | *28* | *23.0- 41.0%* |
|  | Lower limb, bilateral | 44 | *35.2* | *26.9- 44.2%* |  |  |  |  |  |
|  | One upper & one lower limb | 2 | *1.6* | *0.00- 5.7%* |  |  |  |  |  |
| **ADLA Frequency** | |  |  |  | **ADLA Frequency** | |  |  |  |
|  | Never | 37 | *29.6* | *21.8- 38.4%* |  | Never | 68 | *54.4* | *51.0- 70.4%* |
|  | Less than once per month | 59 | *47.2* | *38.2- 56.3%* |  | Less than once per month | 27 | *21.6* | *16.7- 33.4%* |
|  | At least once per month | 26 | *20.8* | *14.1- 28.9%* |  | At least once per month | 15 | *12.0* | *7.8- 21.3%* |
|  | Unknown | 3 | *2.4* | *0.5- 6.9%* |  | Unknown | 1 | *0.8* | *0.02- 4.9%* |
| **Dreyer Stage^1^**  (lower limb only) | |  |  |  | **Stage^2^** | |  |  |  |
|  | 1- 2 | 81 | *66.4* | *55.8- 73.1%* |  | 1 | 23 | *18.9* | *13.6- 29.5%* |
|  | 3+ | 41 | *33.6* | *24.7- 41.8%* |  | 2+ | 88 | *72.1* | *70.5- 86.4%* |
|  |  |  |  |  |  | Unknown | 0 | *0* | *0.0- 3.3%* |
| **Entry lesions** | |  |  |  | **Grade^2^** | |  |  |  |
|  | Present | 59 | *47.2* | *22.5- 39.3* |  | 0 | 97 | *77.6* | *79.7- 92.9%* |
|  | None | 66 | *52.8* | *60.7- 77.5%* |  | 1+ | 13 | *10.4* | *6.4- 19.2%* |
|  |  |  |  |  |  | Unknown | 1 | *0.8* | *0.02- 4.9%* |

^1^ According to the Dreyer system [1]: stage 1 = reversible lymphedema (limb may return to normal, for example at night), stage 2+ = non-reversible lymphedema.
**^2^** According to the staging and grading system of Capuano and Capuano [2]: Stage 1 = smaller than a tennis ball. Grade 0 = No visible burial or shortening of penis

**References**

1. Dreyer G, Addiss D, Dreyer P, Norões J, Rio F. Treatment and Prevention of Problems Associated with Lymphatic Filariasis. Part 2. Tutor's Guide. World Health Organisation; 2001.

2. Capuano G, Capuano C. Surgical management of morbidity due to lymphatic filariasis: the usefulness of a standardized international clinical classification of hydroceles. Trop Biomed. 2012;29(1):24-38.
